# Investigation of the Efficacy and Mechanism of Monoacylglycerol Lipase Inhibitors in Diabetic Foot Ulcers

**DOI:** 10.3390/ph19010171

**Published:** 2026-01-19

**Authors:** Zixia Liang, Ying Wang, Meijia Li, Honghua Li, Yanzhong Han, Yun Zhao, Jian Yang, Yujun Tan, Guoxin Dai, Na Guo, Jingchun Yao, Xiaoyan Lu, Guimin Zhang

**Affiliations:** 1 School of Traditional Chinese Materia Medica, Guangdong Pharmaceutical University, Guangzhou 510006, China; zxliang2026@163.com; 2 School of Medicine and Pharmacy, Ocean University of China, Qingdao 266003, China; wy22932@163.com; 3 School of Chinese Materia Medica, Tianjin University of Traditional Chinese Medicine, Tianjin 301617, China; 4 State Key Laboratory of Integration and Innovation of Classic Formula and Modern Chinese Medicine, Lunan Pharmaceutical Group Co., Ltd., Linyi 276005, China; 15192847565@163.com (H.L.); yanzhong0208@sina.com (Y.H.); zy120418@126.com (Y.Z.); yj7194_cn@sina.com (J.Y.); tyjun1985@163.com (Y.T.); gn150423@163.com (N.G.); yaojingchun@yeah.net (J.Y.)

**Keywords:** monoacylglycerol lipase inhibitor, MAGL11, diabetic foot ulcer, wound healing, angiogenesis, Rap1

## Abstract

**Background/Objectives**: Wound healing proceeds in a timely and sequential manner through four well-defined phases: hemostasis, inflammation, proliferation, and remodeling. To explore the therapeutic efficacy and underlying mechanism of a novel monoacylglycerol lipase (MAGL) inhibitor (designated as MAGL11), a diabetic mouse model of skin wounds was established. **Methods**: Wound healing progression was assessed via gross observation, while histological analyses (including HE staining and Masson staining) were conducted to evaluate tissue repair. Additionally, proteomic analysis and in vitro experiments were employed to validate the therapeutic effects and clarify the molecular mechanism of MAGL11. **Results**: In vivo studies revealed that treatment with MAGL11 significantly accelerated wound closure in diabetic mice. Compared with the control group, MAGL11-treated wounds exhibited notably increased granulation tissue formation and collagen deposition, which was accompanied by a distinct anti-inflammatory effect. Results from proteomic profiling and in vitro experiments further demonstrated that MAGL11 exerted its pro-healing effects by promoting the activation of the Rap1/PI3K/Akt signaling pathway. Specifically, MAGL11 enhanced the migration and chemotaxis of fibroblasts (NIH3T3), human umbilical vein endothelial cells (HUVECs), and keratinocytes (HaCaT) while simultaneously inhibiting cellular apoptosis—all of which collectively contributed to improved wound healing. **Conclusions**: These findings suggest that MAGL11 holds promise as a potential candidate for diabetic wound therapy, primarily through its ability to promote angiogenesis, fibroblast activation, and epithelial regeneration.

## 1. Introduction

Among its complications, Diabetic foot ulcers (DFUs) stand out as one of the most common and severe, and they remain the leading cause of non-traumatic amputations in diabetic patients. Epidemiological data indicate that approximately 15–25% of diabetic patients develop wound ulcers globally [1], and the risk of amputation in diabetic individuals is 10–40 times higher than that in non-diabetic counterparts [2]. In patients with DFUs, at least 17% of such patients are severe enough to require amputation [3,4]. DFUs also increase the risk of all-cause mortality; relevant studies have shown that compared with patients without DFUs, those with the condition have a 22% higher all-cause mortality rate [5,6]. Permanent healing of ulcerative wounds in diabetic patients remains a challenging process, primarily due to hyperglycemia-induced alterations in the wound microenvironment that disrupt the normal recovery phases; consequently, the management of DFUs necessitate a multidisciplinary and multi-faceted therapeutic approach [7,8]. Currently, the main novel strategies employed for DFU treatment include antibiotic therapy, non-invasive vascular testing, debridement surgery, nanomedicine, as well as various dressings integrated with growth factors or stem cells [8,9,10,11], yet each of these methods is associated with distinct limitations: antibiotic therapy can directly and systemically eliminate pathogenic microorganisms to prevent infection spread, but it fails to address the fundamental causes of ulceration and may lead to hepatorenal injury [12]; non-invasive vascular testing, a safe and trauma-free tool for screening arterial diseases, is prone to false-normal results in ankle-brachial index (ABI) measurements, which may mask the actual ischemic status of the affected limb [13]; and various topical dressings (incorporating growth factors or stem cells) can deliver critical growth signals or repair cells to the wound site, yet they suffer from high costs and potential unknown risks [14]. The limitations of current medications for diabetic wound care further underscore the urgent need for more innovative pharmaceutical approaches to treat DFUs.

MAGL11 is a class of compounds that target monoacylglycerol lipase (MAGL)—a key enzyme in the endocannabinoid system [15]. Currently, most MAGL inhibitors (e.g., JZL184, MAGLIN-4) are artificially synthesized small molecules, while the mechanism of action of some lead compounds is inspired by natural regulatory substances involved in the endocannabinoid metabolic pathway [16]. MAGL inhibitors have been demonstrated to possess diverse biological activities [15]. Inhibition of MAGL reduces the degradation of the endocannabinoid 2-arachidonoylglycerol (2-AG), which in turn decreases the release of arachidonic acid (AA) and enhances the activation of cannabinoid type 1 (CB1) and type 2 (CB2) receptors, ultimately exerting neuroprotective, analgesic, anti-inflammatory, and anxiolytic effects [17]. Relevant studies have demonstrated that MAGL inhibitors can alleviate neuroinflammation and amyloid pathology, while also regulating the transformation of microglia from a pro-inflammatory phenotype to a neuroprotective phenotype [18]. The endocannabinoid system exerts regulatory effects on skin homeostasis maintenance and wound healing processes at both the cutaneous local and central nervous system levels [19,20]. Furthermore, cannabinoids may accelerate wound healing by modulating the fibrotic process, promoting re-epithelialization, and alleviating inflammatory responses [21,22].

Despite the abundance of current research on the treatment of DFUs, the translation of preclinical findings into clinical applications remains bottlenecked [23]. This study aims to investigate whether MAGL11 can alleviate the pathological symptoms of DFUs and systematically elucidate its molecular mechanisms underlying the regulation of diabetic wound healing, thereby providing a novel candidate drug and theoretical basis for the clinical treatment of DFUs.

## 2. Results

### 2.1. MAGL11 Promotes Migration and Chemotaxis of HUVECs, HaCaT and NIH3T3 Cells Under High-Glucose Conditions

**Figure 1:**
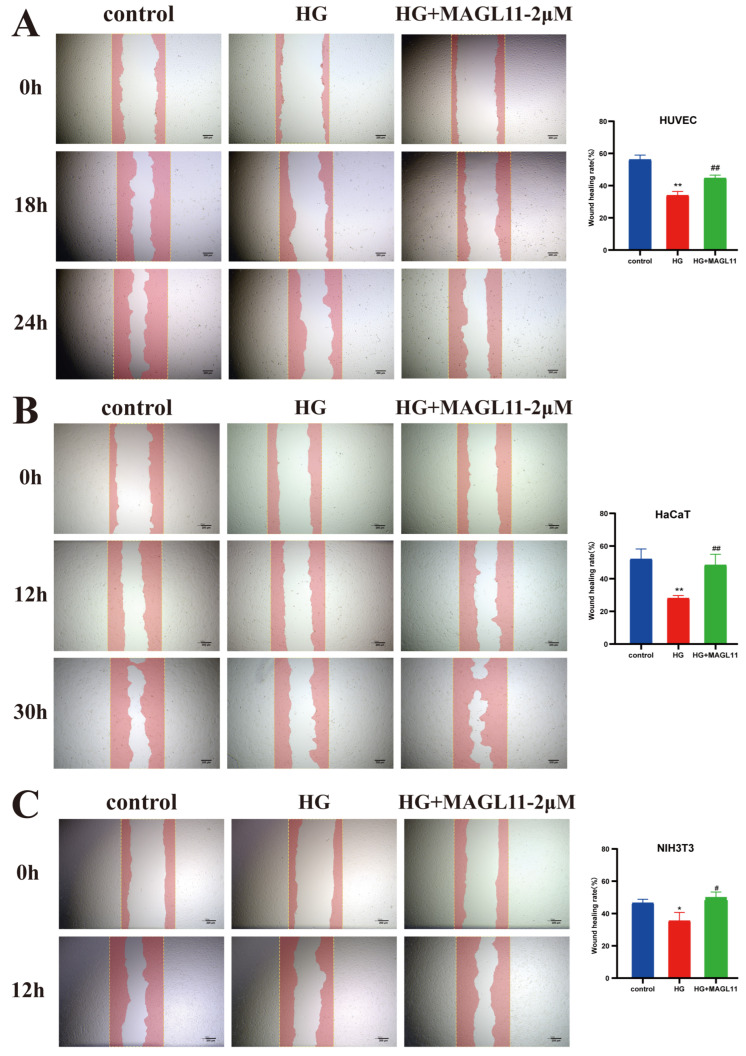
MAGL11 promotes the migration of HUVEC, HaCaT and NIH3T3 cells. (**A**) Cell migration of HUVEC. (**B**) Cell migration of HaCaT. (**C**) Cell migration of NIH3T3. Data were analyzed by one-way ANOVA with Tukey’s post hoc test and presented as mean ± SD. Compared with control: * *p* < 0.05, ** *p* < 0.01; vs. HG group: ^#^ *p* < 0.05, ^##^ *p* < 0.01.

**Figure 2:**
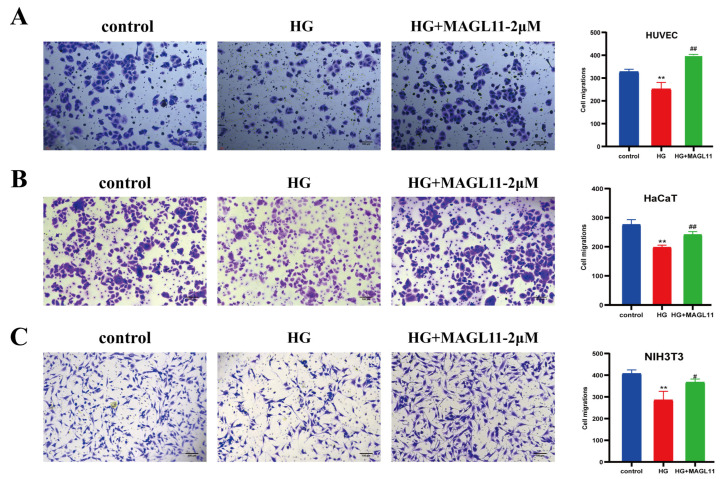
MAGL11 promotes the chemotaxis of HUVEC, HaCaT and NIH3T3 cells. (**A**) Transwell migration experiment of HUVEC. (**B**) Transwell migration experiment of HaCaT. (**C**) Transwell migration experiment of NIH3T3. Data were analyzed by one-way ANOVA with Tukey’s post hoc test and presented as mean ± SD. Compared with control: ** *p* < 0.01; vs. HG group: ^#^ *p* < 0.05, ^##^ *p* < 0.01.

In this study, HUVECs, HaCaT, and NIH3T3 cells under high-glucose (HG) conditions were treated with MAGL11 to investigate its effect on the migration and chemotaxis of these three cell types. As shown in Figure 1A–C, the migration area of the three cell types in the HG + MAGL11-2μM group was larger than that in the HG group. As indicated by crystal violet staining results in Figure 2A–C, after MAGL11 treatment, the number of the three cell types that migrated through the Transwell membrane was significantly higher than that in the diabetic group. All data demonstrated that MAGL11 could promote the migration and chemotaxis of HUVECs, HaCaT and NIH3T3 cells, confirming that MAGL11 can facilitate angiogenesis and epidermal regeneration.

### 2.2. MAGL11 Promotes Proliferation of HUVECs, HaCaT and NIH3T3 Cells Under High-Glucose Conditions

**Figure 3:**
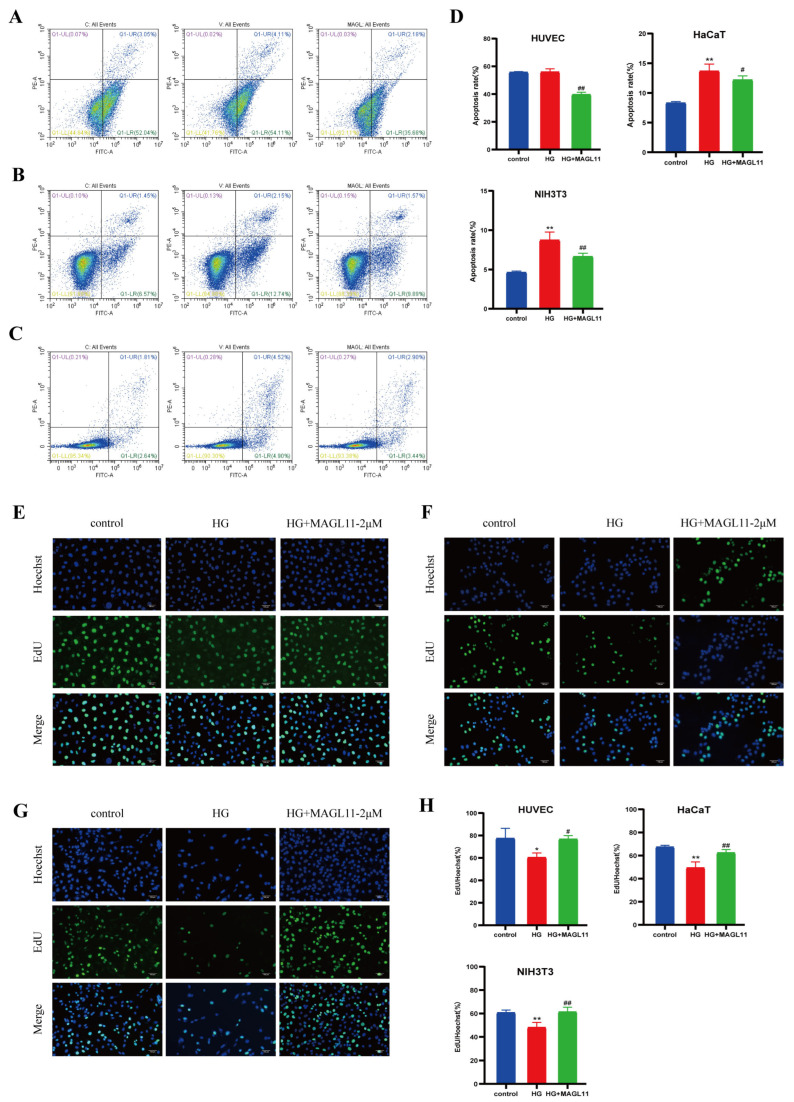
MAGL11 reduces cell apoptosis under high-glucose conditions. (**A**) Flow cytometry for apoptosis detection of HUVEC. (**B**) Flow cytometry for apoptosis detection of HaCaT. (**C**) Flow cytometry for apoptosis detection of NIH3T3. (**D**) Statistical results of apoptotic cells in flow cytometry. (**E**) EdU proliferation staining results of HUVEC. (**F**) EdU proliferation staining results of HaCaT. (**G**) EdU proliferation staining results of NIH3T3. (**H**) Statistical analysis of apoptotic cell percentage based on EdU staining assay results. (**E**–**G**) Scale bar = 100 μm. Data were analyzed by one-way ANOVA with Tukey’s post hoc test and presented as mean ± SD. Compared with control: * *p* < 0.05, ** *p* < 0.01; vs. HG group: ^#^ *p* < 0.05, ^##^ *p* < 0.01.

**Figure 4:**
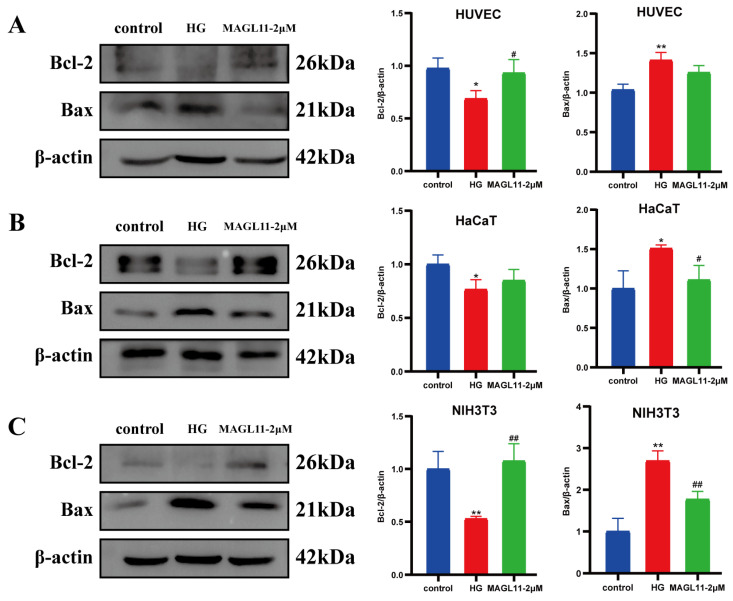
MAGL11 (**A**) Detection of apoptosis-related protein expression in HUVEC by Western blot. (**B**) Detection of apoptosis-related protein expression in HaCaT by Western blot. (**C**) Detection of apoptosis-related protein expression in NIH3T3 by Western blot. Data were analyzed by one-way ANOVA with Tukey’s post hoc test and presented as mean ± SD. Compared with control: * *p* < 0.05, ** *p* < 0.01; vs. HG group: ^#^ *p* < 0.05, ^##^ *p* < 0.01.

HG conditions directly or indirectly inhibit the growth of key cells involved in wound repair, leading to delayed wound healing. As shown in Figure 3A–D (flow cytometry analysis), compared with the HG group, the apoptosis rates of HUVECs, HaCaT and NIH3T3 cells in the HG + MAGL11-2μM group were significantly reduced. EdU assay was used to detect cell proliferation; as presented in Figure 3E–H, the proliferative activity of the three cell types was enhanced after MAGL11 treatment. Western blot results in Figure 4A–C showed that, compared with the HG group, the expression of Bcl-2 protein was increased and the expression of Bax protein was decreased in the three cell types of the HG + MAGL11-2μM group.

### 2.3. Pharmacodynamic Effects of MAGL11 on STZ-Induced DFU Mice

A DFU model was successfully established using 55 mg/kg STZ, confirming the therapeutic effect of topical MAGL11 on diabetic wounds. As shown in Table 1, Mauchly’s test of sphericity yielded a *p*-value > 0.05, confirming the sphericity assumption for time points and thus verifying the reliability of subsequent repeated-measures ANOVA results. A significant main effect of time points indicated an extremely strong influence of time on wound healing; meanwhile, a significant time × group interaction effect suggested that distinct time-dependent patterns of wound healing rates existed among mice in different groups. Further intergroup pairwise comparisons revealed statistically significant differences between the diabetic group and MAGL11-treated groups at all time points. Consistent with Figure 1: on day 3 post-wounding, the wound areas of both the control group and MAGL11-1.28 μM group were significantly reduced; from days 7 to 13, the diabetic group exhibited markedly slower healing than the control group, whereas all MAGL11-treated groups showed varying degrees of improvement in wound healing compared with the diabetic group.

**Figure 5:**
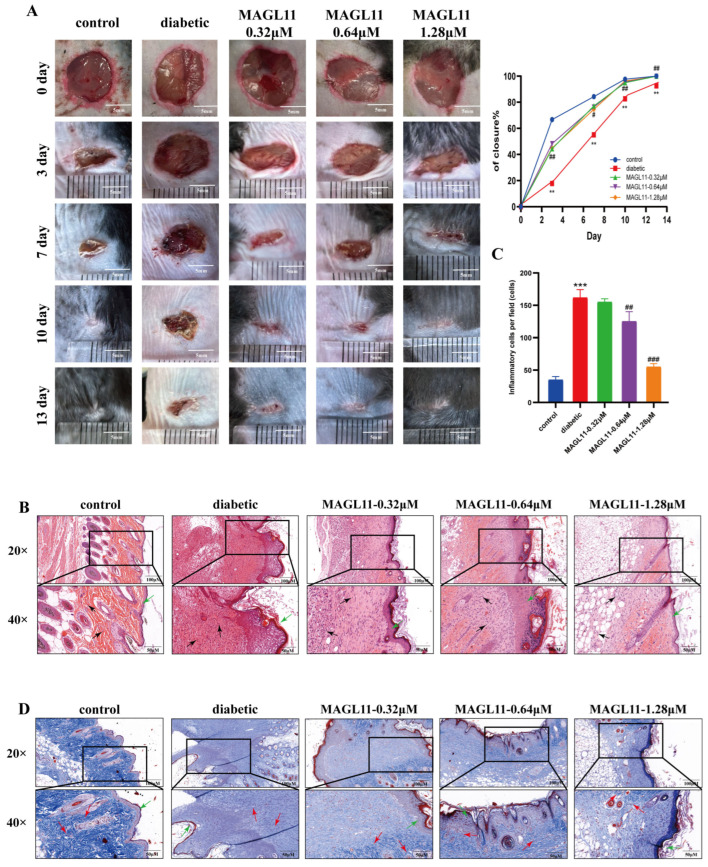
Promotes diabetic wound healing, epidermal regeneration, and collagen synthesis (**A**) Changes in wound area of diabetic mice (n = 8). (**B**) HE-stained pathological images of wound skin tissues in mice (20× and 40×) (n = 8). (**C**) Inflammatory cell count per high-power field (HPF) in HE-stained sections. (**D**) MASSON-stained pathological images of wound skin tissues in mice (20× and 40×) (n = 8). Black arrows indicate inflammatory cells; green arrows denote the epidermal layer; red arrows point to fibroblasts. Data were analyzed by one-way ANOVA with Tukey’s post hoc test and presented as mean ± SD. Compared with control: ** *p* < 0.01, *** *p* < 0.001; vs. diabetic group: ^#^ *p* < 0.05, ^##^ *p* < 0.01, ^###^ *p* < 0.001.

HE staining and Masson staining were used to evaluate wound healing. HE staining clearly revealed the hierarchical structure of wound tissues and inflammatory responses. As shown in Figure 5B,C, the normal group displayed a dense, well-organized epidermal structure with thickened collagen fiber bundles and negligible inflammatory cell infiltration. In contrast, the wounds from the diabetic group presented excessive proliferation of epidermal basal cells coupled with impaired differentiation and maturation. Accumulation of a large number of incompletely differentiated cells within the epidermal layer led to abnormal thickening of the re-epithelialized tissue. Concurrently, the collagen fibers appeared loose and disorganized, with prominent inflammatory cell infiltration detected in the wound area. Compared with the model group, the high-dose MAGL11 treatment group exhibited a tightly arranged epidermis with thickness comparable to that of the normal group, collagen fibers aligned parallel to the skin surface, and a significant reduction in inflammatory cell infiltration. As shown in Figure 5D, collagen was stained blue, with blue-stained areas indicating collagen content. In the normal group, collagen fibers appeared as tightly arranged, orderly, uniformly dark blue bundles of consistent thickness, and spindle-shaped/stellate fibroblasts with dense chromatin were evenly scattered within the fibers. In contrast, the diabetic group showed sparse, lightly stained collagen fibers interwoven into a disorganized meshwork, along with abnormally increased fibroblasts characterized by light cytoplasmic staining, irregular morphology, and reduced activity. Compared with the diabetic group, collagen fibers and fibroblasts in the treatment group were restored to a normal-like state.

### 2.4. Effects of MAGL11 on Cytokines and Growth Factors in Diabetic Wounds

**Figure 6:**
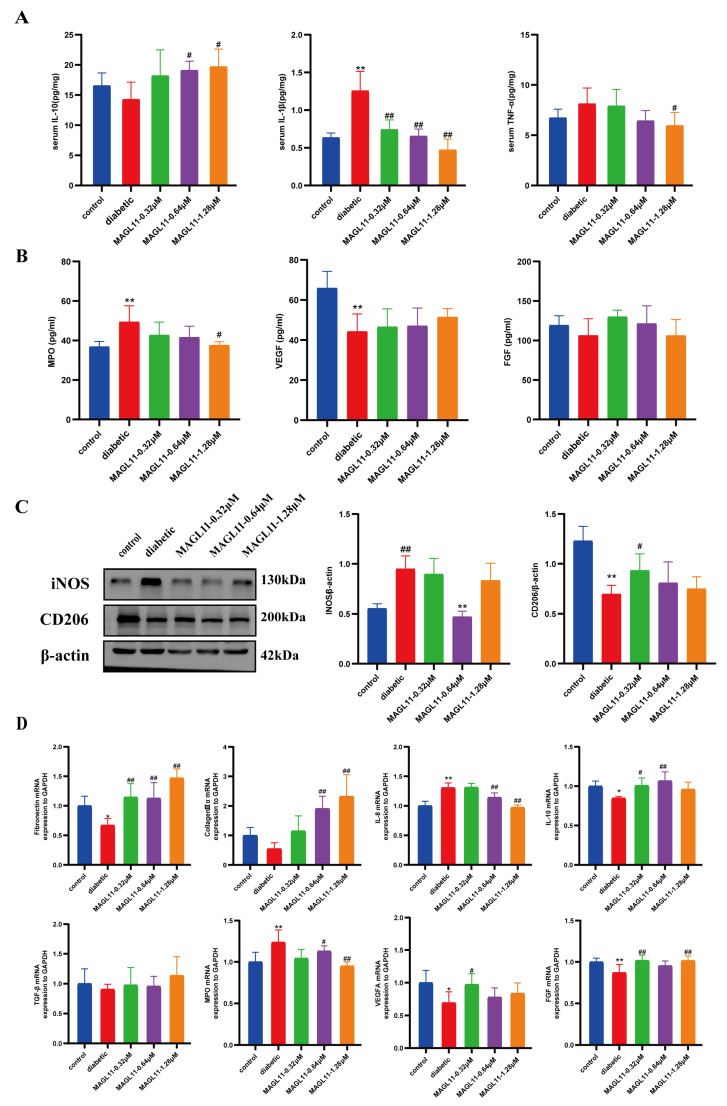
MAGL11 promotes Wound Healing in diabetic mice by regulating the production of inflammatory factors and cytokines. (**A**) Expression levels of IL-10, IL-1β, and TNF-α in serum (n = 6). (**B**) Contents of MPO, VEGF, and FGF in skin tissue supernatants (n = 6). (**C**) Representative images and quantitative analysis of iNOS and CD206 in skin tissues (n = 6). (**D**) Relative gene expression of *FN*, *IL-8*, *ColⅢα*, *IL-10*, *TGF-β*, *MPO*, *VEGFA* and *FGF* in wound tissues on day 13 post-injury (n = 6). Data were analyzed by one-way ANOVA with Tukey’s post hoc test and presented as mean ± SD. Compared with control: * *p* < 0.05, ** *p* < 0.01; vs. diabetic group: ^#^ *p* < 0.05, ^##^ *p* < 0.01.

We detected the secretion of cytokines in serum. As shown in Figure 6A, compared with the control group, the levels of pro-inflammatory factors TNF-α and IL-1β were increased, while the level of anti-inflammatory factor IL-10 was decreased in the diabetic group. After topical application of MAGL11, the levels of these cytokines tended to return to normal compared with the diabetic group. During the proliferative phase, angiogenesis and fibroblasts synergistically promote wound tissue repair and regeneration. As shown in Figure 6B, the supernatant of mouse wound skin tissue was detected using an ELISA kit. Compared with the diabetic group, the normal group showed significantly lower MPO levels and increased VEGF and FGF levels. After topical application of MAGL11, the levels of these factors tended to normalize compared with the diabetic group. INOS is a marker of M1 pro-inflammatory macrophages, and CD206 is a marker of M2 anti-inflammatory macrophages. Western blotting results, as shown in Figure 6C, indicated that compared with the diabetic group, the MAGL11-treated group showed decreased INOS protein expression and increased CD206 protein expression. Meanwhile, qPCR was used to detect the expression of related genes. As shown in Figure 6D, compared with the diabetic group, the MAGL11-treated group exhibited significantly decreased levels of the pro-inflammatory chemokine *IL-8* and *MPO*, and increased levels of the anti-inflammatory chemokines *IL-10*, *TGF-β*, *fibronectin (FN)*, *Collagen (Col)IIIα*, *VEGFA* and *FGF*. Conclusion: MAGL11 can promote the polarization of M1 macrophages to M2 phenotype, exert anti-inflammatory effects, regulate neutrophil activity, and promote the synthesis of growth factors.

### 2.5. The Rap1/PI3K/Akt Signaling Pathway Is the Key Molecular Mechanism Underlying MAGL11-Mediated Diabetic Wound Therapy

**Figure 7:**
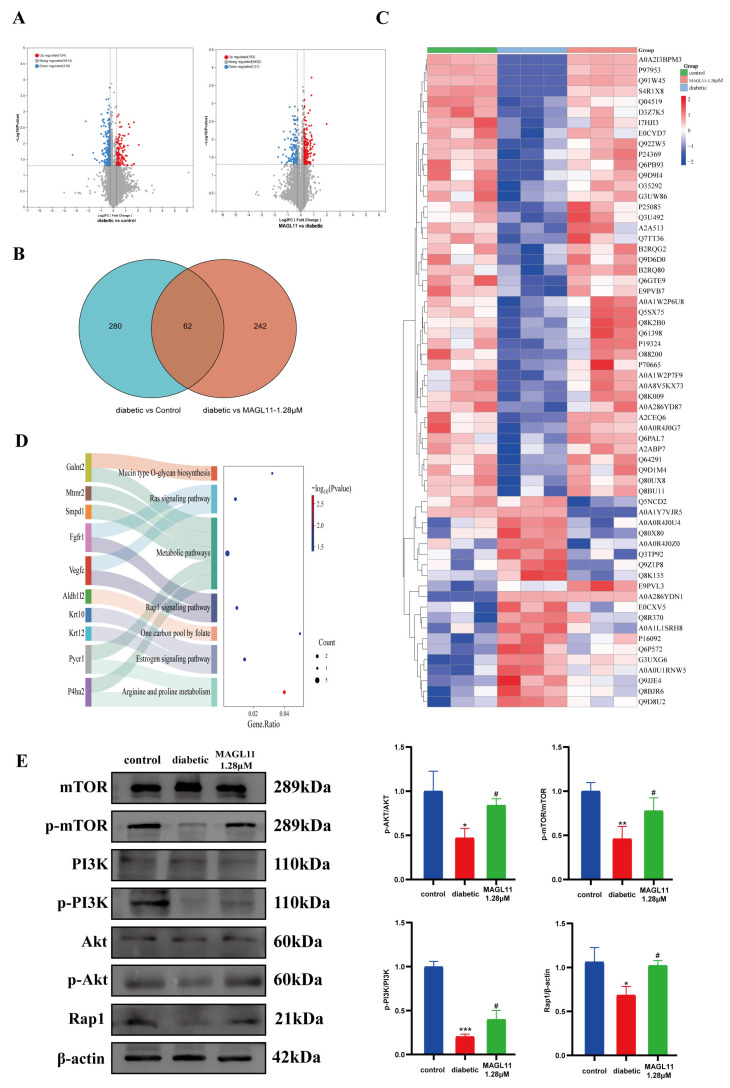
MAGL11 promotes the Rap1/PI3K/Akt pathway in skin tissues. (**A**) Volcano plot. (**B**) Compared with the control group, the diabetic group showed changes in 342 proteins; compared with the model group, the MAGL11 group showed changes in 304 proteins, among which 62 proteins overlapped. (**C**) Heatmap of differential proteins between the three groups. (**D**) KEGG pathway analysis. (**E**) Typical images and quantitative analysis of Rap1, p-PI3K, PI3K, Akt, p-Akt, mTOR, and p-mTOR in mouse skin tissues. Data were analyzed by one-way ANOVA with Tukey’s post hoc test and presented as mean ± SD. Compared with control: * *p* < 0.05, ** *p* < 0.01, *** *p* < 0.001; vs. model group: ^#^ *p* < 0.05.

To investigate the systematic effect of MAGL11 on protein expression in diabetic wound tissues, this study employed a proteomic approach for analysis. Results showed that a total of 9768 proteins were identified in skin tissues. Differentially expressed proteins (DEPs) between groups were screened using the criteria of *p* < 0.05 and Fold Change ≥2 or ≤0.5. The volcano plot in Figure 7A visually illustrates the distribution characteristics of DEPs: compared with the diabetic group, 342 proteins were differentially expressed in the normal group, while 304 DEPs were identified in the MAGL11 group relative to the diabetic group. Notably, as shown in Figure 7B, there were 62 common DEPs between the “diabetic group vs. control group” and “diabetic group vs. MAGL11-1.28 μM group” comparisons. The heatmap in Figure 7C presents the relative expression levels of these DEPs across the three groups.

These proteins were uploaded to the KOBAS database (http://bioinfo.org/kobas; accessed on 3 September 2025) for KEGG pathway enrichment analysis to explore the signaling pathways they regulate. As shown in Figure 7D, in the STZ-induced diabetic wound model, MAGL11 was involved in regulating multiple signaling pathways, including Arginine and Proline Metabolism, Rap1 Signaling Pathway, and Ras Signaling Pathway. Multiple studies have confirmed that the Rap1/PI3K/AKT pathway is a potential therapeutic target for ischemic diseases, tumors, inflammatory vascular diseases, and other conditions.

To verify the above findings, Western blot analysis was further performed to detect the expression of proteins related to the Rap1/AKT/PI3K signaling pathway. For the Rap1 signaling pathway, the results in Figure 7E show that compared with the diabetic group, the expression levels of Rap1, phosphorylated Akt (p-Akt), phosphorylated PI3K(p-PI3K), and phosphorylated mTOR (p-mTOR) proteins were upregulated in the MAGL11 group. This result confirms that MAGL11 can promote the activation of the Rap1 signaling pathway, which is consistent with the proteomic analysis results.

## 3. Discussion

Wound healing requires the coordinated involvement of multiple components, such as blood vessels, fibroblasts, and cytokines, and is mainly divided into four consecutive and overlapping phases: hemostasis, inflammation, proliferation, and remodeling, all of which proceed in an intertwined and sequential manner [24,25]. The detailed preparation method and selection basis of MAGL11, a monoacylglycerol lipase inhibitor used in this study, are available in a published patent (Patent No.: US2023/0090255A1), and the inhibitor can be prepared following the procedure described in Example 1 of this patent. This study draws the following conclusions: (1) MAGL11 can promote wound healing. (2) MAGL11 can enhance angiogenesis and fibroblast proliferation by regulating the Rap1/PI3K/Akt signaling pathway. (3) In in vitro experiments, MAGL11 can promote the proliferation of mouse embryonic fibroblasts (NIH3T3), human umbilical vein endothelial cells (HUVECs), and human immortalized keratinocytes (HaCaT) under high-glucose conditions. Collectively, these results confirm that MAGL11 may accelerate wound healing by exerting anti-inflammatory effects, promoting vascular regeneration and fibroblast proliferation.

The natural wound healing process involves two cell types, neutrophils and macrophages, which play dominant roles in anti-infection and inflammatory regulation during the early stage of wound healing [26,27]. Upon wounding, neutrophils are the first to be activated and migrate to the wound site through the gaps between vascular endothelial cells, followed by the release of pro-inflammatory cytokines such as TNF-α and IL-1β [28,29]. Macrophages then initiate functional responses: they can polarize into pro-inflammatory (M1) or anti-inflammatory (M2) phenotypes in response to distinct signals from the surrounding microenvironment. iNOS is the most characteristic marker of M1 macrophages—when macrophages receive pro-inflammatory signals such as interferon-γ (IFN-γ), they highly express iNOS to exert anti-infective effects [30]. Therefore, reduced levels of pro-inflammatory cytokines, elevated levels of anti-inflammatory cytokines, downregulated iNOS expression, and upregulated CD206 expression can all serve as evidence to support the anti-inflammatory effect of MAGL11.

HUVECs, NIH3T3 and HaCaT were selected to establish an in vitro model, as these cell types correspond to the three core processes of diabetic wound healing—angiogenesis, granulation tissue formation, and re-epithelialization [31,32,33]. Specifically, HUVECs represent a classic model for assessing angiogenic efficacy [34], HaCaT cells act as the core functional cells mediating epidermal repair [35], and NIH3T3 cells govern granulation tissue formation and remodeling by regulating wound contraction [33]. Collectively, these three cell types form a dynamically coordinated repair network that underpins the entire trajectory of wound healing from injury onset to complete resolution [36]. In vitro experimental results demonstrated that MAGL11 intervention markedly enhanced the proliferative activity (assessed by EdU-positive rates), Transwell chemotactic migration capacity, and scratch wound closure rate of all three cell types. Concurrently, flow cytometry analysis confirmed a significant reduction in cell apoptosis. These findings indicate that MAGL11 exerts its pro-healing effects through three complementary mechanisms: (1) enhancing HUVEC proliferation and migration to provide a sufficient cellular source for capillary network assembly, thereby delivering oxygen and nutrients to fibroblasts and keratinocytes; (2) promoting NIH3T3 proliferation and chemotaxis to ensure sustained granulation tissue formation, which serves as a structural scaffold for the migration of the other two cell types; (3) improving HaCaT functional activity to accelerate wound re-epithelialization, protect nascent tissues, and ultimately terminate the repair process. In conclusion, MAGL11 can concurrently ameliorate the proliferation, migration, and anti-apoptotic functions of these three cell types in diabetic wounds, synergistically driving the healing process via multiple key regulatory nodes. This combined cell model fully recapitulates the multi-cellular regulatory features inherent to physiological wound healing, and its reliability has been validated by relevant published studies [37].

Proteomic profiling and Western blot analyses in this study indicated that MAGL11 may exert a therapeutic effect on diabetic wound healing by promoting activation of the Rap1/PI3K/Akt signaling pathway. A wealth of published research has confirmed that this pathway orchestrates multiple physiological processes, including cell survival, proliferation, migration, metabolism, and angiogenesis. Specifically, Rap1 plays a pivotal role in cell adhesion, polarization, and migration by modulating integrin affinity and intracellular trafficking [38]. More importantly, Rap1 serves as a key upstream activator of the PI3K/Akt pathway: activated Rap1 can directly enhance the catalytic activity of PI3K, which in turn catalyzes the conversion of phosphatidylinositol 4,5-bisphosphate (PIP2) to phosphatidylinositol 3,4,5-trisphosphate (PIP3); PIP3 then recruits Akt to the cell membrane, triggering Akt activation [39]. By inhibiting the hydrolysis of 2-AG, MAGL11 elevates intracellular 2-AG levels, which subsequently fully activates its cognate receptors—particularly CB2—thereby exerting anti-inflammatory effects and fundamentally optimizing the microenvironment for angiogenesis. Concurrently, CB2 activation can trigger PI3K pathway activation, followed by the sequential activation of the Akt and Rap1 signaling cascades [40]. Western blot band analysis revealed that the protein expression levels of Rap1, p-PI3K, and p-Akt were upregulated, suggesting that the Rap1/PI3K/Akt signaling pathway may be involved in mediating the pro-healing effect of MAGL11.

MAGL11 enables precision-targeted therapy via topical administration, which markedly elevates local 2-AG levels at the wound site and thus synchronously coordinates the regulation of multiple key processes during wound healing. However, the present study has several notable limitations. First, female mice were not included in the experiments, which may restrict the applicability of the findings to male populations and preclude the direct extrapolation of intervention effects to female individuals. Second, the mechanistic investigation lacks sufficient depth, as targeted validation experiments were not performed to confirm whether the Rap1/PI3K/Akt pathway is an indispensable downstream target mediating the therapeutic effects of MAGL11. Third, given that this is a short-term observational study, the long-term safety and efficacy of MAGL11 remain elusive.

Despite these limitations, the current study still provides valuable insights for future research on DFU treatment and identifies clear directions for subsequent investigations. Specifically, future studies may conduct targeted validation experiments to further elucidate the mechanism underlying MAGL11-mediated therapeutic effects on diabetic wounds. Moreover, exploring the potential systemic side effects and compensatory mechanisms induced by long-term administration will be a key step in advancing the clinical development of this agent.

## 4. Materials and Methods

### 4.1. Chemicals and Reagents

MAGL11 (N-(3-chlorophenyl)-1-(3-hydroxybenzoyl) piperidine-4-formamide) (prepared according to the method in patent US 2023/0090255 A1, purity > 99%). Dimethyl sulfoxide (DMSO; product code: 12191502). Streptozotocin (STZ; product code: V900890) was purchased from Sigma-Aldrich (St. Louis, MO, USA) and freshly dissolved in sterile cold 0.1 M citrate buffer prior to each injection.

### 4.2. Experimental Animals

Eighty male C57BL/6J mice, weighing 20–22 g and aged 6–8 weeks, were purchased from Jinan Pengyue Laboratory Animal Breeding Co., Ltd. (Jinan, China, License No.: SCXK (Lu) 2023-0023). The mice were housed in a specific-pathogen-free (SPF) environment under controlled conditions of temperature (20–26 °C), relative humidity (40–70%), and a 12 h light/12 h dark cycle, with free access to irradiated sterilized full-nutrient pellet feed. This study was approved by the Experimental Animal Care and Use Committee of Lunan Pharmaceutical Group Co., Ltd. (Approval No.: HN-IACUC-2025-023). All animal experiments were conducted in accordance with the Guide for the Care and Use of Laboratory Animals. The protocol was approved on 13 March 2025.

### 4.3. Cell Culture and Treatment Procedures

Human umbilical vein endothelial cells (HUVEC), human keratinocytes (HaCaT), and fibroblasts (NIH3T3) were cultured in high-glucose Dulbecco’s Modified Eagle Medium (DMEM). Specifically, 10% fetal bovine serum (FBS) was added to the culture medium for HUVECs and HaCaT cells, while 10% newborn calf serum (NBCS) was supplemented into the medium for NIH3T3 cells. All cells were cultured in an incubator with 5% CO_2_ at 37 °C. For in vitro experiments, the cells were divided into three groups: the control group, the 100 mM glucose group (HG), and the 100 mM glucose + 2 μM MAGL11 group (HG + MAGL11-2 μM).

### 4.4. Wound Healing Assay

Cells were seeded into 24-well plates at an appropriate density and cultured until reaching 90% confluence. Parallel scratches were made in the center of each well using the tip of a 200 μL pipette tip. The wells were then rinsed twice with phosphate-buffered saline (PBS) to remove detached cell debris, followed by the addition of respective drug treatments. Cell images were captured under a microscope at different time points, and the scratch area was measured using ImageJ 1.8.0 software [41].

### 4.5. Transwell Assay

DMEM containing 10% serum was added to the lower chambers of 24-well Transwell inserts. Cells cultured in serum-free DMEM were seeded into the upper chambers. After 24 h of culture, the medium in both upper and lower chambers was replaced with high-glucose DMEM, and respective drug treatments were added to the lower chambers. Following 12 h of incubation in the incubator, the inserts were rinsed twice with PBS, fixed with 4% paraformaldehyde for 15 min, and stained with crystal violet for 20 min. Excess crystal violet was thoroughly washed off with PBS, and cells on the upper surface of the insert membranes were wiped away with cotton swabs. The inserts were then observed under a microscope, and the number of chemotactic cells was quantified using ImageJ software [42].

### 4.6. Annexin V-FITC/PI Assay

Cells were seeded into 24-well plates and cultured overnight for 24 h to allow adherence. After drug treatment for another 24 h, the supernatant was collected into flow cytometry tubes. The adherent cells were rinsed twice with PBS, then digested with EDTA-free trypsin, and the digested cells were also collected into flow cytometry tubes. All operations were performed on ice. The cells were centrifuged for 10 min. According to the kit instructions, 500 μL of Binding Buffer, 5 μL of FITC-, and 5 μL of PE-PI were added to the cells, followed by incubation for 10 min in the dark. Finally, the samples were detected using a flow cytometer, and the data were analyzed with CytoFlex 1.2 software [43].

### 4.7. EdU Assay

Cells were cultured in 24-well plates until reaching 50% confluency, then incubated at 37 °C with high-concentration glucose and drugs. Appropriate concentrations of EdU solution were added according to cell type, followed by continued culture. Subsequently, cells were fixed with 4% paraformaldehyde, permeabilized with 0.5% Triton-X100, then incubated with Click reaction solution in the dark for 30 min, observed under a fluorescence microscope, and analyzed using ImageJ.

### 4.8. Establishment of a Diabetic Mouse Model

Eighty mice were stratified-randomized by body weight into 5 groups (n = 16 per group): control group, diabetic group, the low-dose MAGL11 group (MAGL11-0.32 μM), the medium-dose MAGL11 group (MAGL11-0.64 μM), and the high-dose MAGL11 group (MAGL11-1.28 μM). The control group was raised without modeling; the diabetic and treatment groups received 55 mg/kg STZ intraperitoneally daily for 5 days. On day 9 post-last STZ injection, mice with random blood glucose >16.7 mmol/L were considered diabetic. These modeled mice were then block-randomized (block size = 4): sorted by ascending glucose, 4 mice with similar levels formed a block, and each block was allocated to the three treatment groups and the diabetic groups via a random number table. This study omitted separate vehicle and citrate buffer groups given validated buffer inertness, adherence to the 3R principle and consistency with established protocols [44]. Based on an effect size of f = 0.6 from the pilot experiment, a power analysis was performed using G.Power 3.1.9.7 software (α = 0.05, power = 80%). With a 20% dropout rate reserved, 16 mice were assigned to each group to meet the requirements of statistical tests and multiple comparisons.

### 4.9. Establishment of Wound Model and Administration of Therapeutic Agents

All mice were anesthetized via intramuscular injection of 1.5 mg/mL tiletamine-zolazepam (Shutai) and 1.5 mg/mL xylazine (Sailazine); their dorsal skin hair was shaved, and two full-thickness wounds (1 cm in diameter, extending to the fascia layer) were created using sterile instruments. Treatments were initiated on the day of wound creation: no treatment was administered to mice in the normal control group; each wound of mice in the diabetic group was topically treated with 5 μL of DMSO; and each wound of mice in the three MAGL11 treatment groups was topically administered with 5 μL of MAGL11 solution at concentrations of 0.32 μM, 0.64 μM, and 1.28 μM, respectively, with the above treatments continued for 5 consecutive days. The dosage and administration schedule of MAGL11 were determined collectively based on the in vitro cell safety concentration window, body surface area normalization method, and in vivo pre-experiments. A double-blind method was used: materials and animals were coded, operators and analysts remained blinded, and unblinding was conducted after the experiment.

### 4.10. Detection of Wound Healing Rate

Photographs starting from day 0 after wound modeling, photographs were taken on days 0, 3, 7, 10, and 13 to document the wound healing status [45]. The wound area was analyzed using ImageJ software. Data analysis was performed with SPSS 26.0. Repeated–measures ANOVA was used to compare wound closure rates across groups and time points; the Greenhouse–Geisser correction was applied when sphericity was violated. For significant interaction effects, data were stratified by time points, and pairwise comparisons were conducted via one–way ANOVA with Bonferroni correction. Statistical significance was set at α = 0.05 (*p* < 0.05). The difference in wound healing rates between the normal group and the diabetic group was analyzed using an independent-sample *t*-test. Line graphs were generated using GraphPad Prism 9.0. All mice were euthanized on day 13 after wound modeling; part of the wound tissue was preserved in 4% paraformaldehyde, another part was stored in −80 °C cryotubes, and mouse serum samples were stored at −80 °C.

### 4.11. Histopathology

On day 13, dorsal skin wounds were excised from the dorsal skin of mice. Following fixation in 4% paraformaldehyde for 24 h, paraffin sections were prepared and subjected to HE and Masson staining, respectively. These sections were subjected to morphological observation and semi-quantitative counting analysis to evaluate the degree of inflammatory cell infiltration and collagen fiber formation.

### 4.12. Enzyme-Linked Immunosorbent Assay (ELISA) Kit

Mouse skin tissues were harvested, minced, and lysed in phosphate-buffered saline (PBS). After centrifugation, the supernatant was collected. The levels of myeloperoxidase (MPO), vascular endothelial growth factor A (VEGFA), and fibroblast growth factor (FGF) in the mouse skin tissues were detected in accordance with the instructions provided with ELISA [46].

### 4.13. Determination Using U-PLEX Mouse Cytokine Detection Kit (MSD)

After mice were anesthetized, abdominal aortic blood was collected. The blood samples were centrifuged at 4000 rpm for 10 min to separate the serum. In accordance with the kit instructions, the serum samples were added to MSD plates pre-coated with capture antibodies, followed by the addition of detection antibodies and then reading buffer. Finally, the plates were placed into an MSD reader to measure the electrochemiluminescence signal intensity of each well, and the levels of tumor necrosis factor (TNF)-α, interleukin (IL)-1β, and IL-10 in mouse serum were determined [47].

### 4.14. RT-qPCR

Total RNA was extracted from mouse wound skin tissues using a commercial reagent, and complementary DNA (cDNA) was synthesized therefrom. Glyceraldehyde-3-phosphate dehydrogenase (GAPDH) was used as the internal reference gene. The primer sequences for each target gene are listed in Table 2. Quantitative real-time polymerase chain reaction (qPCR) was performed on a 7500/96 Fast Real-Time PCR System (96-well) using Power SYBR Green PCR Master Mix (Thermo Fisher Scientific, Waltham, MA, USA), following the standard protocol. The relative expression level of target gene mRNA was calculated using the comparative threshold cycle (Ct) method, with the formula: 2^−∆∆Ct^ [48].

### 4.15. Western Blotting

Western blotting was employed to detect total proteins in skin tissues and cell pellets. In the experiment, mouse wound skin tissues and cell pellets were first lysed using a protein extraction kit purchased from Beyotime Institute of Biotechnology (Haimen, China). and the total protein concentration was determined with a BCA protein assay kit. After separation by 12% sodium dodecyl sulfate-polyacrylamide gel electrophoresis (SDS-PAGE), 50 μg of protein samples were transferred onto polyvinylidene fluoride (PVDF) membranes. The membranes were blocked with 5% non-fat milk for 2 h, then incubated with primary antibodies at 4 °C overnight, followed by incubation with horseradish peroxidase (HRP)-conjugated anti-mouse or anti-rabbit secondary antibodies. Finally, protein bands were visualized using a Chemi Scope 6200 automatic chemiluminescence imaging system (Shanghai Qinxiang Science Instruments Co., Ltd., Shanghai, China), and the gray values of the bands were analyzed with ImageJ software [49].

### 4.16. Proteomics Research

All experimental procedures related to proteomics, including sample pretreatment, protein extraction, enzymatic hydrolysis, mass spectrometry detection, and raw data acquisition, were performed according to the method described by Sun et al. [50]. The raw data were preprocessed and initially analyzed using the Central Cloud Platform (Cloud.MajorBio.com, a free online analysis platform). Statistical analysis between groups was conducted using the *t*-test function in the R language to calculate the significant *p*-value and fold change (FC). Differentially expressed proteins (DEPs) were defined with the screening criteria of *p* < 0.05 and |FC| > 2, where upregulated proteins were identified as FC > 2 and downregulated proteins as FC < 0.5. Furthermore, the Kyoto Encyclopedia of Genes and Genomes (KEGG; https://www.genome.jp/kegg/; accessed on 25 October 2025) pathway database was used to perform functional enrichment analysis on DEPs, aiming to explore the core metabolic pathways they participate in and their potential biological functions [51].

### 4.17. Statistical Analysis

Statistical analysis was performed using GraphPad Prism 9.0 software. All measurement data were expressed as mean ± standard deviation (x ± s). Comparisons between two groups were analyzed by unpaired *t*-test, and comparisons among multiple groups were conducted using one-way analysis of variance (ANOVA) followed by Tukey’s post hoc test. A *p*-value < 0.05 was considered statistically significant.

## 5. Conclusions

In conclusion, our research findings indicate that MAGL11 exerts a significant therapeutic effect on wound healing in diabetic mice. This study demonstrates that such a therapeutic effect is achieved by activating the Rap1/PI3K/Akt pathway while promoting the proliferation and migration of HUVECs, HaCaT, and NIH3T3 cells. Therefore, MAGL11 holds potential as a therapeutic agent for DFUs.

## Figures and Tables

**Table 1:** Wound closure rates and statistical analysis results of mice in different groups at each postoperative time point ($\bar{x}$ ± s, %) (n = 8).

Group	3 Day	7 Day	10 Day	13 Day
diabetic	18.81 ± 4.52	56.36 ± 5.17	82.39 ± 5.34	92.52 ± 3.78
MAGL11 0.32 μM	50.36 ± 3.58 ^##^	74.83 ± 2.26 ^##^	95.01 ± 5.12 ^##^	99.55 ± 1.02 ^##^
MAGL11 0.64 μM	51.47 ± 4.09 ^##^	76.20 ± 4.85 ^##^	95.70 ± 3.76 ^##^	99.84 ± 0.35 ^##^
MAGL11 1.28 μM	48.31 ± 5.72 ^##^	75.18 ± 5.63 ^##^	95.20 ± 3.87 ^##^	99.73 ± 0.64 ^##^
Intergroup F-value	109.422	29.165	15.485	27.296
Intergroup *p*-value	<0.001	<0.001	<0.001	<0.001

Note: Mauchly’s Test of Sphericity: Mauchly’s W = 0.880, χ^2^ = 3.412, *p* = 0.637. Significant main effect of time points: F = 2012.599, *p* < 0.001, η^2^ = 0.996. Significant time × group interaction effect: F = 23.992, *p* < 0.001, η^2^ = 0.72. Intergroup *p*-value: compared with the model group at the same time point, all dose groups showed *p* < 0.001. Compared with diabetic: ^##^ *p* < 0.01.

**Table 2:** Nucleotide sequences of target genes.

*Gene*	*Source*	Forward (5′-3′)	Reverse (5′-3′)
*CollagenIII* *α*	*Mouse*	ACGTAAGCACTGGTGGACAG	AGCTGCACATCAACGACATC
*fibronectin*	*Mouse*	GCTCAGCAAATCGTGCAGC	CTAGGTAGGTCCGTTCCCACT
*IL-8*	*Mouse*	CAAGGCTGGTCCATGCTCC	TGCTATCACTTCCTTTCTGTTGC
*IL-10*	*Mouse*	GCTCTTACTGACTGGCATGAG	CGCAGCTCTAGGAGCATGTG
*TGF-β*	*Mouse*	CTCCCGTGGCTTCTAGTGC	GCCTTAGTTTGGACAGGATCTG
*VEGFA*	*Mouse*	CTGCCGTCCGATTGAGACC	CCCCTCCTTGTACCACTGTC
*MPO*	*Mouse*	AGTTGTGCTGAGCTGTATGGA	CGGCTGCTTGAAGTAAAACAGG
*FGF1*	*Mouse*	CCCTGACCGAGAGGTTCAAC	GTCCCTTGTCCCATCCACG

## Data Availability

The original contributions presented in this study are included in the article. Further inquiries can be directed to the corresponding authors.
